# Lung nodule malignancy classification with associated pulmonary fibrosis using 3D attention-gated convolutional network with CT scans

**DOI:** 10.1186/s12967-023-04798-w

**Published:** 2024-01-13

**Authors:** Yucheng Liu, Hao Yun Hsu, Tiffany Lin, Boyu Peng, Anjali Saqi, Mary M. Salvatore, Sachin Jambawalikar

**Affiliations:** 1 Department of Radiology, Columbia University Irving Medical Center, 3-124B Milstein Hospital Bldg, 177 Fort Washington Avenue, New York, NY 10032 USA; 2 Department of Pathology, Columbia University Irving Medical Center, New York, NY USA

## Abstract

**Background:**

Chest Computed tomography (CT) scans detect lung nodules and assess pulmonary fibrosis. While pulmonary fibrosis indicates increased lung cancer risk, current clinical practice characterizes nodule risk of malignancy based on nodule size and smoking history; little consideration is given to the fibrotic microenvironment.

**Purpose:**

To evaluate the effect of incorporating fibrotic microenvironment into classifying malignancy of lung nodules in chest CT images using deep learning techniques.

**Materials and methods:**

We developed a visualizable 3D classification model trained with in-house CT dataset for the nodule malignancy classification task. Three slightly-modified datasets were created: (1) nodule alone (microenvironment removed); (2) nodule with surrounding lung microenvironment; and (3) nodule in microenvironment with semantic fibrosis metadata. For each of the models, tenfold cross-validation was performed. Results were evaluated using quantitative measures, such as accuracy, sensitivity, specificity, and area-under-curve (AUC), as well as qualitative assessments, such as attention maps and class activation maps (CAM).

**Results:**

The classification model trained with nodule alone achieved 75.61% accuracy, 50.00% sensitivity, 88.46% specificity, and 0.78 AUC; the model trained with nodule and microenvironment achieved 79.03% accuracy, 65.46% sensitivity, 85.86% specificity, and 0.84 AUC. The model trained with additional semantic fibrosis metadata achieved 80.84% accuracy, 74.67% sensitivity, 84.95% specificity, and 0.89 AUC. Our visual evaluation of attention maps and CAM suggested that both the nodules and the microenvironment contributed to the task.

**Conclusion:**

The nodule malignancy classification performance was found to be improving with microenvironment data. Further improvement was found when incorporating semantic fibrosis information.

**Supplementary Information:**

The online version contains supplementary material available at 10.1186/s12967-023-04798-w.

## Introduction

Low-dose computed tomography (CT) is the gold standard for early lung cancer detection [1–3]. These scans are designed to detect lung nodules, which are then determined by radiologists to be either benign or malignant. Currently, lung nodules are characterized according to the Lung CT Screening Reporting and Data System (Lung-RADS) developed by the American College of Radiology (ACR) [4]. This system focuses on information derived from the nodules alone (e.g. solidity, shape, growth rate, and texture) with little consideration given to the nodule microenvironment. Studies, however, have shown that nodule microenvironments such as fibrosis may play a role in lung cancer malignancy. Karampitsakos et al*.* and Li et al*.* found that pulmonary fibrosis increases a patient’s risk of developing lung cancer. Salvatore et al*.* revealed that pulmonary fibrosis is observed in 6.6% of lung cancer screening participants compared to 0.03% among men and 0.02% among women of the general public [5–7]. Many risk factors are shared between pulmonary fibrosis and lung cancer and CT imaging has played a crucial role in the early detection of both diseases [8, 9]

Deep learning (DL) algorithms, specifically convolutional neural networks (CNNs), have been used in lung nodule detection, segmentation, and classification tasks with high accuracy and efficiency [10–17]. An explainable model can be achieved with visualization maps attached to the model, specific features and structures can be highlighted in the images. This could help researchers to better understand how a model reaches decisions, and identify features associated with it [18, 19]. Zhu et al*.* [11] retain the segmented nodules by discarding the background information when performing nodule classification, and Xiao et al*.* [12] isolate nodules from the encompassing tissues based on the presupposition that the presence of surrounding tissue might negatively impact the classification outcome. Other studies [13–17] encompassed adjacent soft tissue structures due to the utilization of 2D or 3D bounding boxes as training data. However, these studies did not conduct a performance comparison between solitary nodules and nodules situated within diverse pulmonary contexts, therefore, the potential impact on the microenvironment still remains unclear. To the best of our knowledge, no DL-based studies have investigated the impact of the fibrotic microenvironment on the lung nodule classification task.

The purpose of this study is to assess the influence of integrating a fibrotic microenvironment aspect into the classification of nodule malignancies through the utilization of DL algorithms. We developed a novel 3D classification network featuring attention gates, specifically designed to mitigate the loss of model efficiency in the processing of nodules and their associated microenvironment. This innovation incorporates several novel technical contributions: (1) multiple attention gates emanate from distinct network depths, serving the purpose of accentuating salient features at shallower network depths. This collective input significantly influences the final classification outcome; and (2) predictive vectors derived from these attention gates can be concatenated with additional clinical data. Subsequently, this combined information is passed through a fully connected layer, culminating in the generation of a definitive classification result. By iteratively incorporating and excluding microenvironmental data in experiments, our investigation aims to elucidate any conceivable associations between fibrotic tissue presence and the categorization of nodule malignancy.

## Materials and methods

Data were retrospectively collected in compliance with the Health Insurance Portability and Accountability Act, institutional review board approval, and waivers of informed consent.

### Study design

**Fig. 1:**
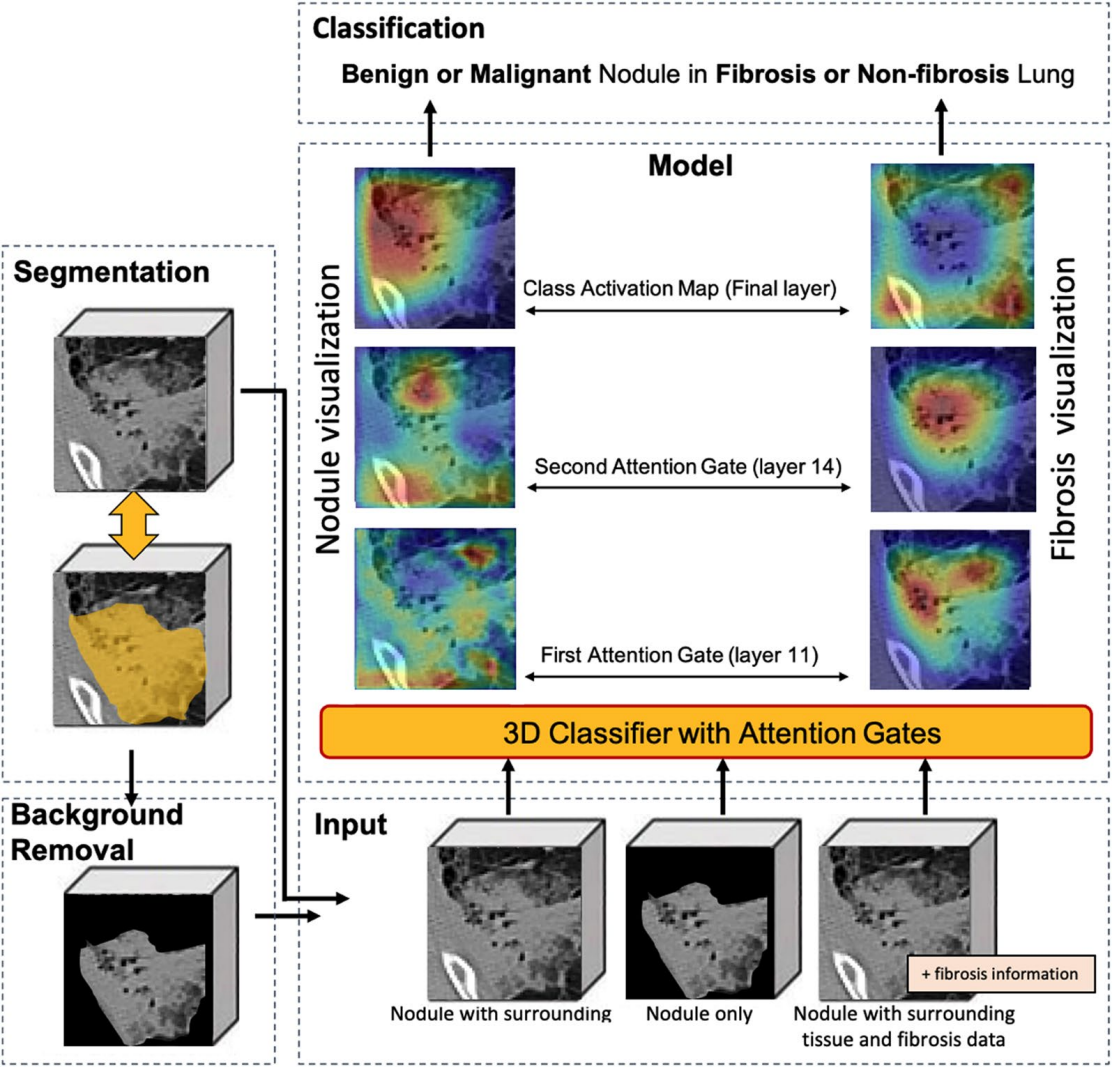
The workflow. Models to segment and classify nodules based on their central point coordinates provided by radiologists. CT images were cropped into 64 × 64 × 64 voxel volumes according to the center coordinates and fed into the models. Nodule volumes are passing (1) the 3D UNet for nodule segmentation. With the segmentation mask, surrounding soft tissue (background) can be removed; hence the nodule volume estimation can be performed. Nodule volumes then go through (2) the classification model (3D Attention Net) to predict nodule malignancy and pulmonary fibrosis. Separate datasets (with or without semantic fibrosis information) can be selected as the input to the classifier. The model’s attention at different layers can be visualized and interpreted via attention coefficient maps and CAMs

4500 patients with chest CT scans containing fibrosis were identified. Patients less than 21 years old at the time of the initial CT scan were excluded. Lung nodules were identified and labeled with a single point marked approximately in the center of the nodule by a senior radiologist with over 20 years of experience via the VGG image annotator (VIA) web-based interface [20]. Two 3D DL models, one for nodule segmentation and one for nodule classification were used in this study (Fig. 1). The segmentation model was trained to delineate nodules. The classification model was trained with the objective of distinguishing between cancerous and non-cancerous nodules, while also determining the presence of fibrotic tissues in the surrounding region. Biopsy results and radiologist reports were used as the ground truth labels. The classification accuracy was compared to the nodule volume doubling time (VDT).

### Datasets

The two datasets used in this study were the publicly available LIDC-IDRI with thoracic CT scans (1018 cases) and the in-house dataset (1088 cases). Information about the in-house dataset can be found in Table 1. The LIDC-IDRI dataset was used for the nodule segmentation task and pre-training of the nodule classification model [21]. We leveraged the high-quality consensus nodule annotations provided in the LIDC dataset to train the segmentation model and the pretraining of the classification model. For the in-house dataset, screening and diagnostic CT examinations were collected from 4500 patients. A pool of 1088 cases met the inclusion and exclusion criteria. Among 1088 cases, 144 cases (13.23%) were confirmed to be malignant by pathological biopsy, and 57 cases (5.24%) were deemed “highly suspicious” by radiologists. For the nodule malignancy classification, we considered nodules in the two scans independently. A dataset containing 345 malignant (labeled 1) and 743 benign (labeled 0) nodules was constructed. Three separate data subsets were used in the training: (1) nodules only (microenvironment removed), (2) nodules with surrounding soft tissue microenvironment, and (3) nodules and microenvironment with semantic fibrosis metadata. For the pulmonary fibrosis classification task, 489 cases out of 1,088 cases were labeled by radiologists as fibrosis. Fibrosis is defined as fibrotic tissue presented adjacent to the nodule or presented in the nodule-growing microenvironment.

**Table 1:**
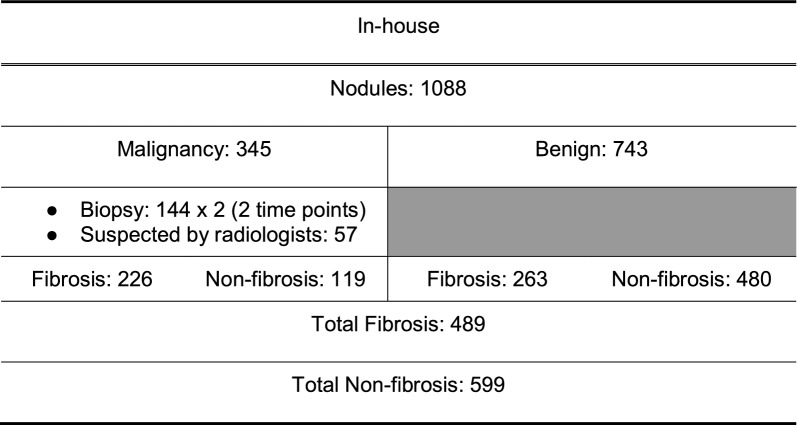
Dataset information

### Segmentation model

The segmentation model within our pipeline served the purpose of preparing inputs for the nodule classification. In this stage, the model generated two distinct datasets: one that excluded background information and another that exclusively included background information. The underlying assumption was that by eliminating the background, any lung fibrosis-related details were either unavailable or significantly minimized. Consequently, the classification model was tasked with making decisions solely based on the attributes of the nodules themselves.

The architecture of our 3D segmentation model was based upon the U-Net framework (3D UNet, Monai [22]), featuring an analysis path (encoder) and a synthesis path (decoder). Within this framework, we incorporated 3D convolutional layers and 3D pooling layers to extract intricate features from the input volume. In the decoding phase, 3D deconvolution was employed to reinstate the original feature map dimensions. Notably, the inclusion of residual units played a pivotal role in enabling the model to encompass contextual information surrounding the lung nodules. By incorporating these residual units, the entire network exhibited heightened precision, consequently ameliorating the segmentation of lung nodules within medical images.

### Nodule malignancy classification

#### Volume doubling time (VDT)

For cases with follow-up CT scans, the nodule volume doubling time (VDT) was calculated. We used our segmentation model to estimate nodule volume. We then used the modified Schwartz formula to calculate VDT: ${\text{VDT}} = \frac{{Ln\left( 2 \right)*{\text{Days Between Scans}}}}{{Ln\left( {Volume\, on\, {\text{Second Scan}}/ {\text{Volume on Baseline Scan}}} \right)}}$ [23]. This formula assumes an exponential growth rate for lung cancer. It assumes that malignant cells divide at a constant rate, and thus lesions grow exponentially with time. It is worth noting that a shrinking nodule will have a negative VDT value. Shrinking nodules were classified as benign in this study. According to most studies, a volume doubling time below 400 days represents a high likelihood of malignancy, and a VDT above 500 days is characteristic of a benign nodule [24]. To prevent high false-positive rates, we set the threshold of malignancy to 500 days.

#### DL-based classification model

**Fig. 2:**
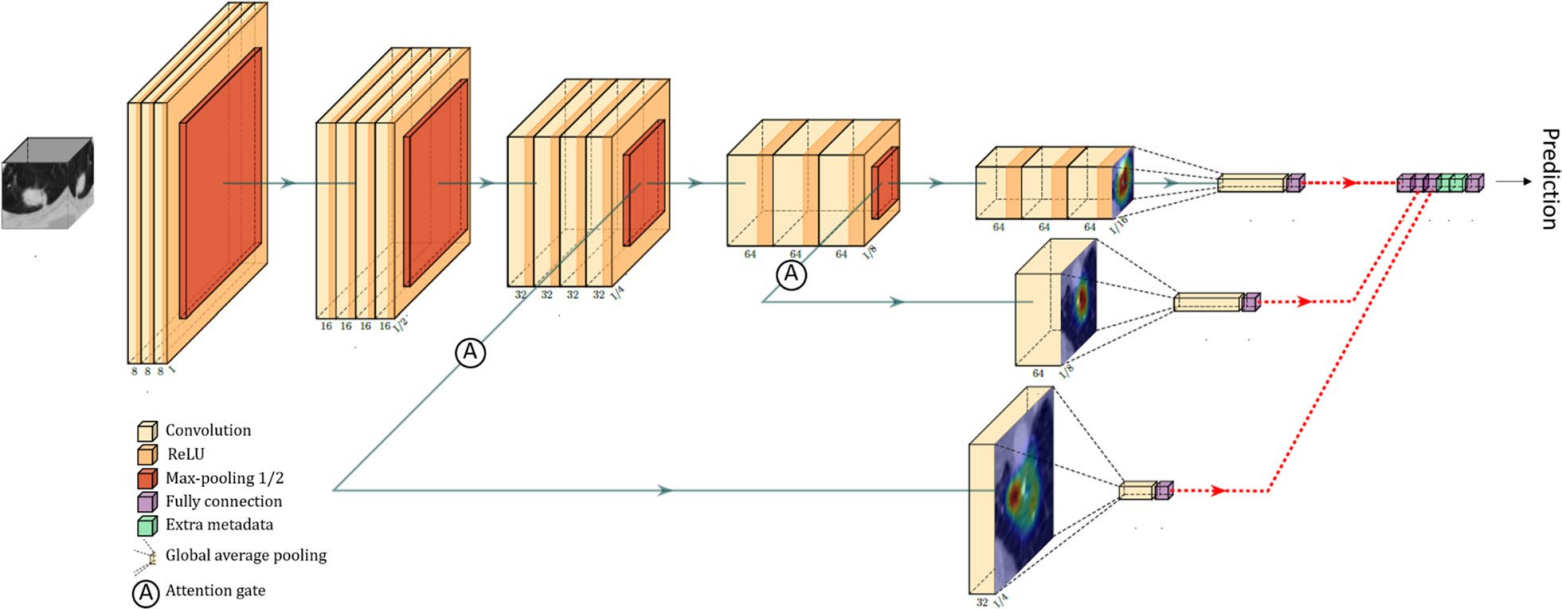
The schematics of the 3D Attention Network. The light-yellow blocks and the dark-yellow blocks indicate the convolutional layers and the ReLU, respectively. The orange blocks indicate the max-pooling operation resulting in a 1/2 image size. The purple blocks indicate the fully connected layer, and the green blocks indicate additional clinical metadata available. The green arrow-line and the red dashed-arrow-line are connecting operations and concatenating vectors, respectively

**Fig. 3:**
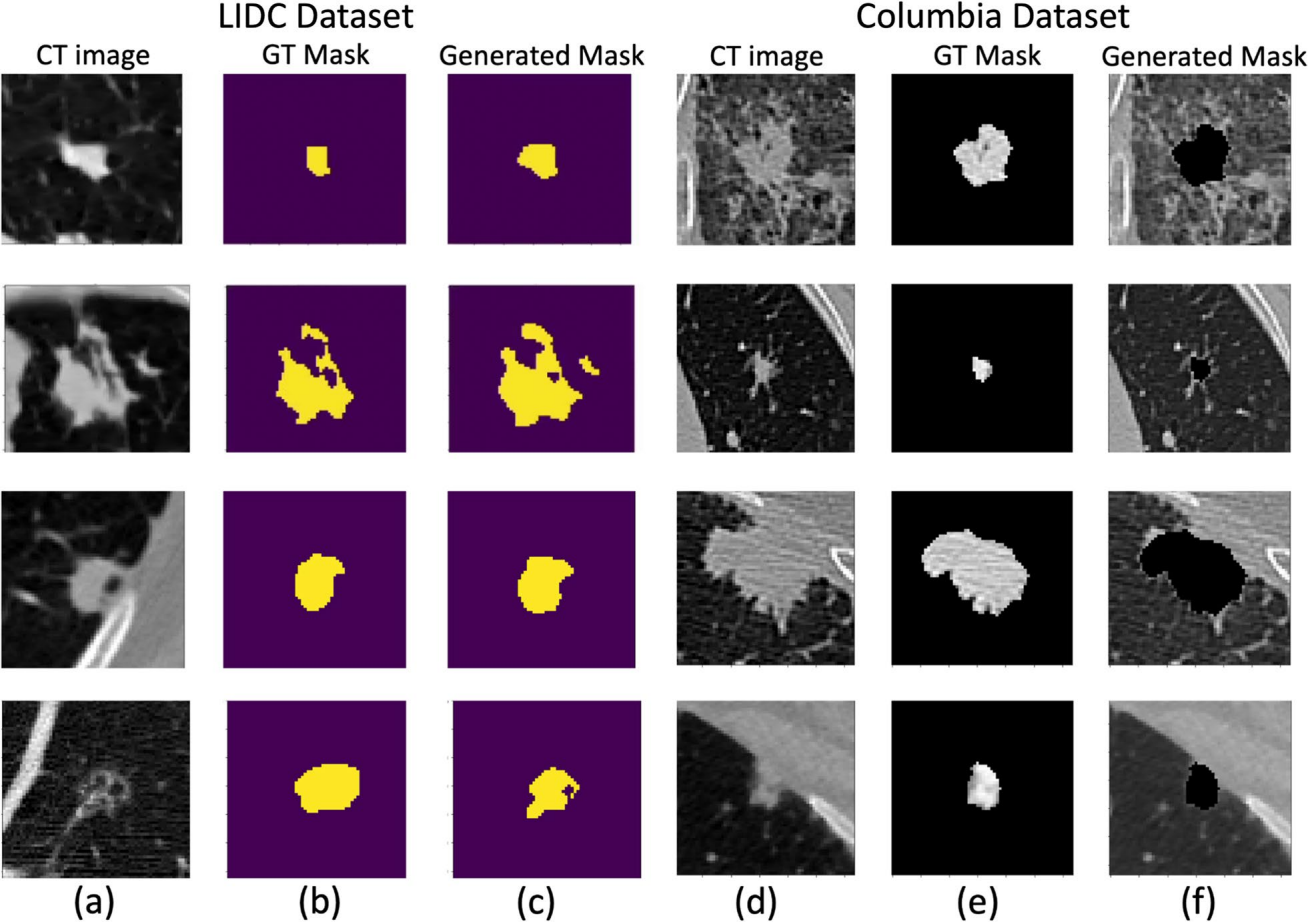
Example cases for nodule segmentation. Four cases from the LIDC dataset and four In-house datasets were randomly selected and displayed here. The LIDC data with radiologist hand-drawn annotations for training and testing and the In-house data were used for inference only. For the LIDC dataset, columns (**a**), (**b**), and (**c**) are the original CT images, ground-truth segmentation provided by radiologists, and the generated masks by segmentation model, respectively. For the In-house dataset, columns (**d**), (**e**), and (**f**) are the original CT images, background removed (nodule only) images via auto-segmentation, and the background only (soft lung tissue) images, respectively

The proposed 3D Attention-gated Network (3D AG-Net) classification model was adopted from the 2D Attention-Gated Sononet [25]. The 3D AG-Net consists of 17 3D convolution layers (3 × 3 × 3 convolution) to extract features from volumetric CT images input. Two soft attention gates, AG-1 and AG-2, were placed at the 11th and 14th layers, respectively (Fig. 2). Deviating from the main network flow, AG-1 and AG-2 can intercept salient features they deem necessary and filter out those features at their specific depths to make independent predictions. The attention maps generated by the AGs provide visualizations of the network behavior as well. Examples of the given nodules are shown in Fig. 3**.**

Hu et al*. *[15] demonstrated that network depths could affect prediction performance on the LIDC dataset, a trade-off between sensitivity and specificity was observed. Higher sensitivity was achieved at a deeper network, and higher specificity was achieved at an intermediate network. Hence, two attention-gated feature extraction modules were branched out at the 11th layer and 14th layer (intermediate depth) from the leading architecture (green lines with attention gates in Fig. 2) to optimize specificity performance. Meanwhile, the leading architecture has 17 layers to provide sufficient depth for improving sensitivity performance. At the final stage of the network, as indicated by red dashed lines in Fig. 2, predictive vectors generated by AG-1, AG-2, the final layer of the leading network, and semantic clinical metadata (e.g., lung fibrosis) can all be concatenated, followed by a fully connected layer to fine-tune the vectors and yield the optimized final prediction results.

All experiments including preprocessing, developing, and evaluation of the model, were performed using Python version 3.6 and PyTorch 1.5 on NVIDIA GP102, GTX 1080 Ti.

### Evaluations and statistical analysis

A tenfold cross-validation strategy was used to evaluate network performance and generalizability. True positive cases, true negative cases, false positive cases, and false negative cases are denoted as TP, TN, FP, and FN respectively. The quantitative evaluation metrics used are listed below..

1 $${\text{Accuracy}} = \frac{{{\text{TP}} + {\text{TN}}}}{{{\text{TP}} + {\text{TN}} + {\text{FP}} + {\text{FN}}}}$$

2 $${\text{Sensitivity}} = \frac{{{\text{TP}} }}{{{\text{TP}} + {\text{FN}}}}$$

3 $${\text{Specificity}} = \frac{{{\text{TN}}}}{{{\text{TN}} + {\text{FP}}}}$$

4 $${\text{Dice Score}} = \frac{{{2 } \times {\text{the Area of Overlap}}}}{{\text{the total number of pixels in both images}}}$$

Qualitative evaluations were conducted with attention maps and class activation maps (CAM). A detailed description of CAM can be found in Additional file 1: Appendix SB. The one-way ANOVA pairwise t-test was performed with a confidence level set at 95%. In the process of evaluating multiple pairwise performances, a heightened susceptibility to committing a Type I error is observed. In order to mitigate this, a corrective measure known as the Bonferroni correction Eq. (5) was applied on the α level restricting a more confined threshold for the adjusted p-value to reject the null hypothesis.

5 $${\text{Bonferroni-corrected p value}} = \frac{{\text{The original p value}}}{{\text{The number of tests performed}}}$$

## Results

### Nodule segmentation

We observed a smooth decrease in the training loss with a fluctuated mean dice score in the validation loss due to the overfitting of the model. Therefore, we include a dropout rate of 20% during each epoch. Example cases of the segmentation results are shown in Fig. 3 for the LIDC dataset and the in-house dataset. We were able to see the similarity between the ground truth and the generated mask. We achieved an average dice score of 0.761 on the validation dataset after 50 epochs of training. The ADAM optimizer was used with the learning rate and dropout rate as 0.0001 and 0.2, respectively. Data augmentation, including random flips, intensity scaling, and intensity shifting, were used to improve model performance. The dice loss function was used as a metric to access the model performance and evaluate the testing dataset. The sliding window inference method which uses a rectangular or cube region of fixed dimension that "slides" across an image was applied to create binary classifications on whether or not each voxel belongs to a nodule. Although computationally expensive, this method determines if the window has an object that interests us. Accelerated methods such as cache IO and transform function featured by MONAI were also used to expedite the training process.

All experiments including preprocessing, developing, and evaluation of the model, were performed using Python version 3.6 and PyTorch 1.5 on NVIDIA GP102, GTX 1080 Ti.

### 3D AG-Net Pre-training on LIDC-IDRI

To ensure the effectiveness of the 3D AG-Net for the nodule malignancy classification task, we validated the classification model using the LIDC dataset, which has public benchmarks. The model was trained with an early stopping strategy with patience of 50 epochs based on the highest validation accuracy. The network used binary cross-entropy (BCE) loss for nodule malignancy classification. The input data is a 64 × 64 × 64 voxel CT nodule image, Adam optimizer with an initial learning rate set at 0.0002, batch size 128. All experiments were conducted on an Nvidia GTX 1080 Ti GPU with PyTorch library. We achieved 91.57%, 83.34%, 90.46%, and 0.95 for accuracy, sensitivity, precision, and AUC, respectively, with the S1 and S2 (benign) versus S4 and 5 (malignant), which is compatible with the state-of-the-art models' performances (Additional file 1: Appendix SC). The result trained with the complete LIDC dataset, S1, S2, S3 (benign) versus S4 and S5 (malignant), also reported here as 85.11%, 77.78%, 88.54%, and 0.90 ± 0.04 for accuracy, sensitivity, specificity, and AUC, respectively (Table 2). Although the model trained with the complete LIDC dataset achieved lower metrics scores, it serves as a better initializer for the training of the in-house dataset since the complete dataset better reflects the real-world conditions. Therefore the model is more generalized for later applications.

**Table 2:** Quantitative results

Dataset	LIDC-IDRI	In-house	In-house		
Training strategy/data processing	NA	NA	Pretrained on LIDC-IDRI data	Pretrained on LIDC-IDRI data/Nodule surrounding tissues removed	Pretrained on LIDC-IDRI data/semantic fibrosis data added
Micro-environment information	Available	Available	Available	Not available	Available
Accuracy (%)^*^	85.11 (3.19)	78.84 (5.88)	79.03 (2.97)^*^	75.61 (7.02)^*^	80.84 (3.31)^*^
Sensitivity (%)^†^	77.78 (12.24)	62.00 (13.65)	65.46 (18.64)^†^	50.00 (25.46)^†^	74.67 (14.78)^†^
Specificity (%)^§^	88.54 (5.87)	87.29 (5.98)	85.86 (6.29)^§^	88.46 (7.88)^§^	84.95 (5.43)^§^
AUC	0.90 (0.04)	0.83 (0.03)	0.84 (0.06)	0.78 (0.08)	0.89 (0.05)

Data are presented in the format of Mean (Standard deviation), *AUC* area under the receiver operating characteristic

*One-way ANOVA analysis performed with Bonferroni correction on accuracy: p-value = 0.0011 (statistical significance)

^†^One-way ANOVA analysis performed with Bonferroni correction on sensitivity: p-value = 0.0013 (statistical significance)

^§^One-way ANOVA analysis performed with Bonferroni correction on specificity p-value = 0.35556 (statistical non-significance)

### 3D AG-Net training on the in-house dataset

**Fig. 4:**
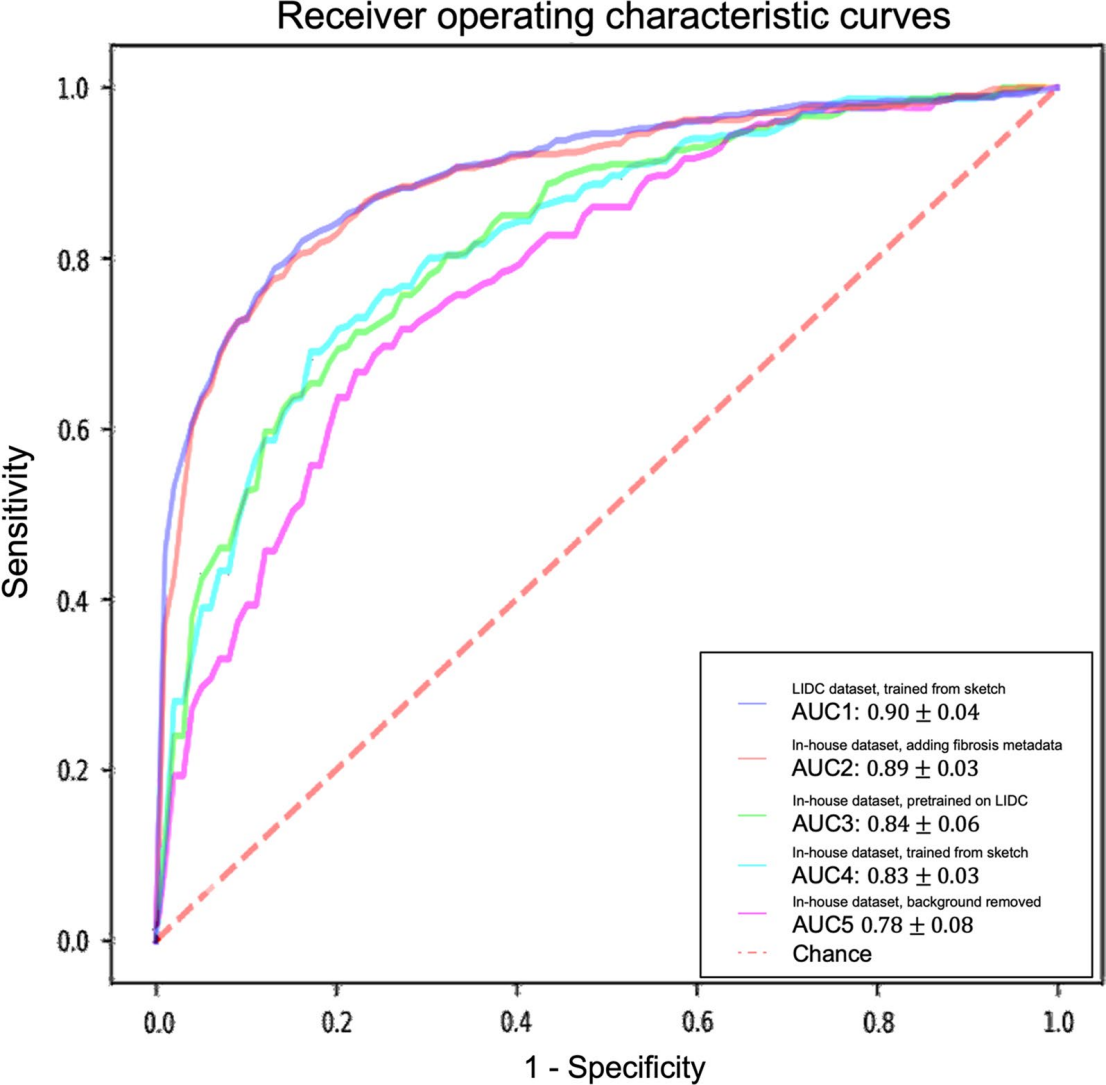
Receiver operating characteristic (ROC) curves and area under the curves (AUC) of experiments with different datasets and methods. The ROC curves demonstrated here are the averaged ROC based on the tenfold cross-validation. The averaged AUCs with one standard deviation are computed and listed in the legend area. The blue line, red line, green line, cyan line, magenta line, and the red dashed-line are indicating the nodule malignancy prediction results on the LIDC dataset, In-house dataset with metadata, In-house dataset (pretrained with LIDC), In-house dataset, and In-house dataset (background removed), respectively. Statistical differences were found in **1** LIDC dataset, trained from sketch v.s. In-house dataset, trained from sketch (p-value: 0.0319); **2** LIDC dataset, trained from sketch v.s. In-house dataset, background removed (p-value: 0.0001); **3** In-house dataset, adding fibrosis metadata v.s. In-house dataset, background removed (p-value: 0.0002)

For models trained with the in-house dataset with tenfold cross-validation, the experiment results are summarized in Table 2 and Fig. 4. The performance of the 3D AG-Net without pretraining (trained from scratch) achieved 78.84 ± 5.88%, 62.00 ± 13.65%, 87.29 ± 5.98%, 0.83 ± 0.03 for accuracy, sensitivity, specificity, and AUC, respectively. The model with pretraining on LIDC dataset achieved results of 79.03 ± 2.97%, 65.46 ± 18.64%, 85.86 ± 6.29%, 0.84 ± 0.06 for accuracy, sensitivity, specificity, and AUC, respectively. When the background information was removed, it performed slightly worse in most of the metrics except for specificity, achieving 75.61 ± 7.02%, 50.00 ± 25.46%, 88.46 ± 7.88%, 0.78 ± 0.08 for accuracy, sensitivity, specificity, and AUC, respectively. When additional semantic fibrosis metadata was provided, the 3D AG-Net yielded the best AUC when comparing all other types of datasets. The result was 80.84 ± 3.31%, 74.67 ± 14.78%, 84.95 ± 5.43%, 0.89 ± 0.05 for accuracy, sensitivity, specificity, and AUC, respectively.

We used 3D AG-Net without pretraining as the baseline to compare with the model pretrained on LIDC dataset (AUC increase 1.2%), the model trained with background removed (AUC decrease 6%, p-value < 0.01), and model trained with additional semantic fibrosis metadata (AUC increase 7.2%, p-value < 0.01). We found statistical differences (p-value < 0.01) in AUC between adding (model trained with additional semantic fibrosis metadata) and removing (model trained with nodule background removed) fibrosis information. When semantic fibrosis information is available to the network, the nodule malignancy classification accuracy increases 5.23%. Nodule volume doubling time (VDT) as the current clinical guideline to predict nodule malignancy is also included in the experiment for comparison. The VDT method achieved 62.63%, 56.52%, and 65.48% of accuracy, sensitivity, and specificity respectively. Note that CNN trained with nodule-only images still outperformed the VDT method.

The 3D AG-Net was also trained with the in-house dataset to predict lung fibrosis using the ground-truth fibrosis diagnosis provided by radiologists (fibrosis as 1, non-fibrosis as 0). The model achieved 73.01 ± 5.84%, 75.18 ± 13.32%, 70.83 ± 6.22%, and 0.75 ± 0.07 for accuracy, sensitivity, specificity, and AUC, respectively.

### Visualization

**Fig. 5:**
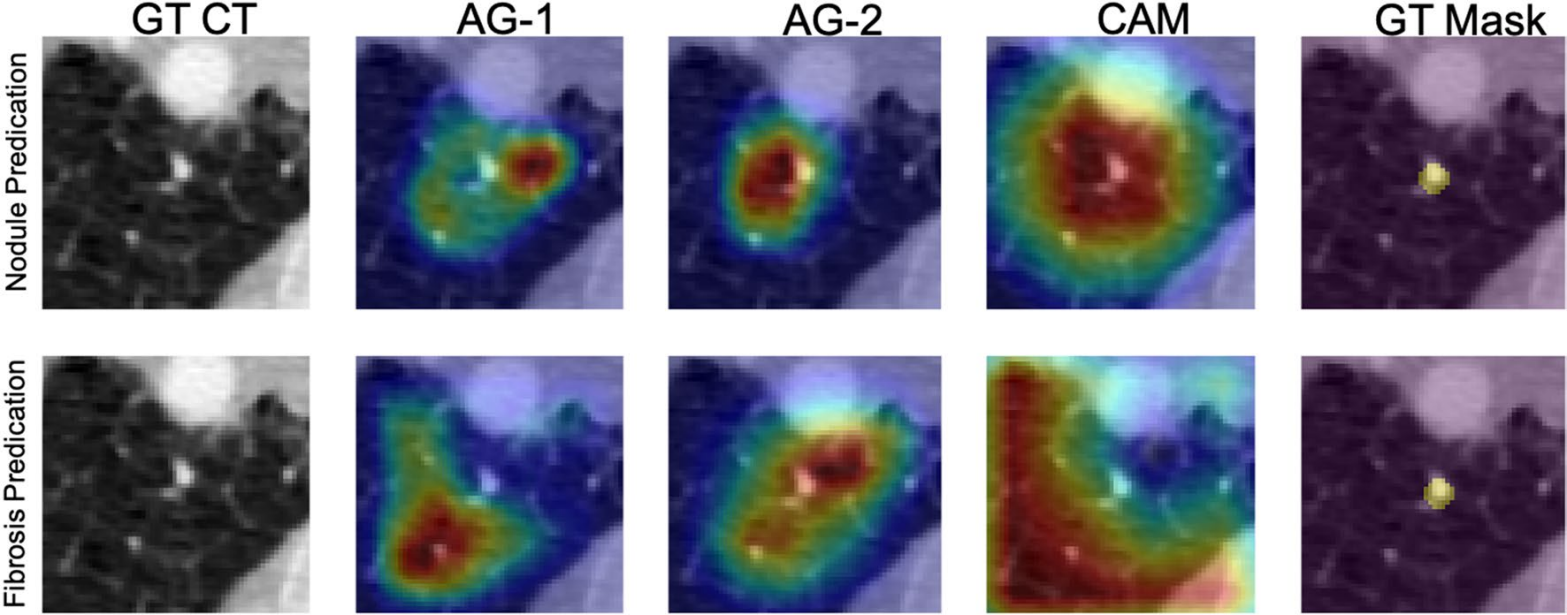
Network attention gates (AGs) and class activation maps (CAMs) visualizations. An example is network visualization for nodule prediction (first row) and lung fibrosis prediction (second row) tasks. The first, second, third, and fourth columns indicate the ground-truth (GT) CT image, first attention gate (AG-1) at No. 11 layer depth, second attention gate (AG-2) at No. 14 layer depth, and the class activation map (CAM) at the final layer, respectively. The fifth column indicates the nodule ground-truth mask (GT Mask), which is not available when the model was trained. The case demonstrated here is a benign nodule in the non-fibrotic lung, where both nodule malignancy and fibrosis models made the correct inferences. From the AGs and CAMs, we can observe the nodule network focuses on nodule parenchyma and its surrounding tissues, while the fibrosis network focuses on other lung tissue with the nodule parenchyma excluded

Figure 5 depicts network visualizations for nodule and fibrosis classification. Notably, distinct attention patterns emerge within the same case when different objectives, such as nodule or fibrosis classification, are employed.

**Fig. 6:**
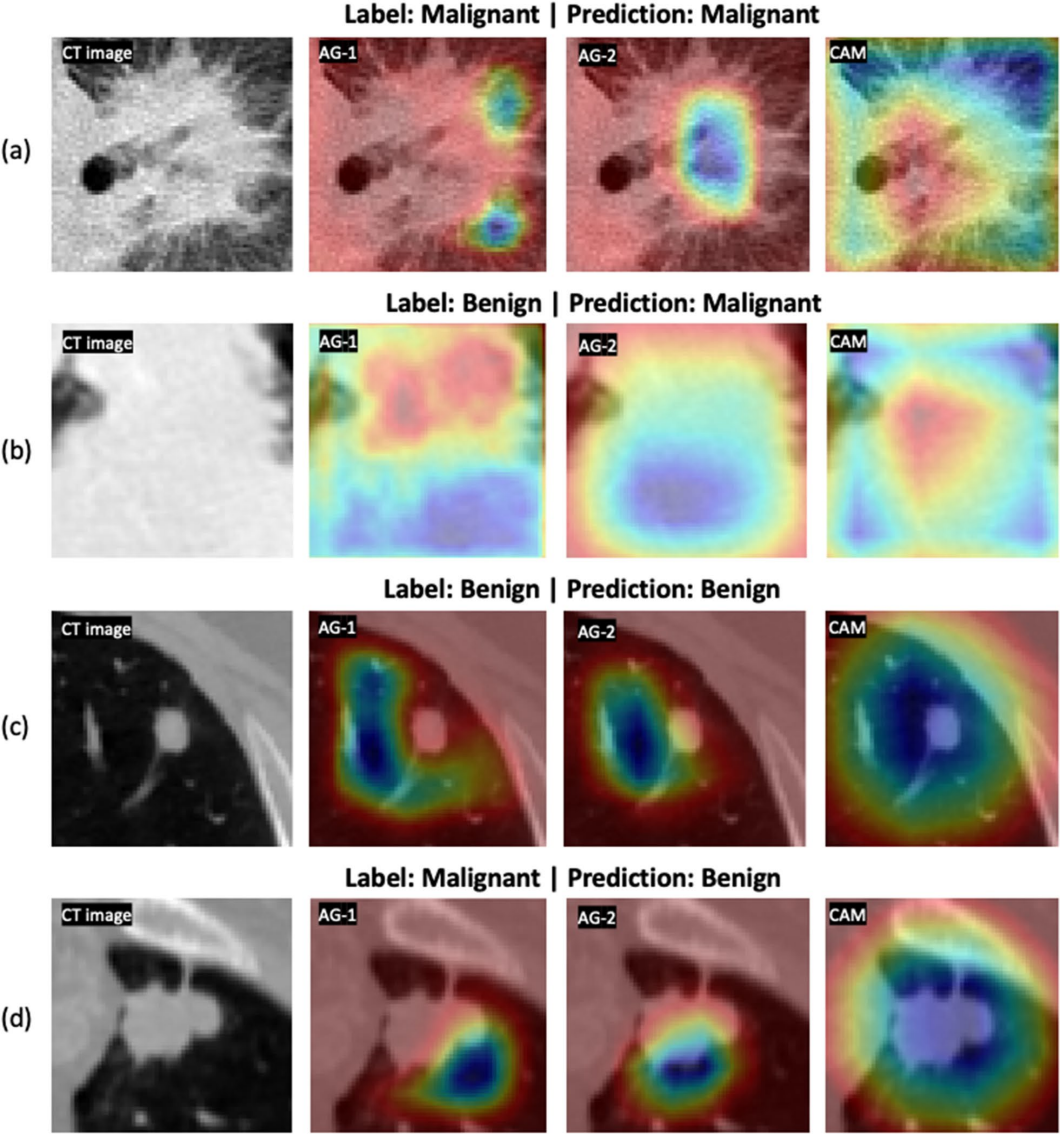
Results of the 3D AG-Net on the in-house dataset. A true positive case (**a**), a false positive case (**b**), a true negative case (**c**), and a false negative case (**d**) were shown. In each case, it showed the center slice of the 64 × 64 × 64 volume, the slice with the AG-1 heatmap, the slice with the AG-2 heatmap, and the slice with CAM on top of it from left to right, respectively

For nodule classification, the network exhibits a similar attention pattern for true positive cases (Fig. 6a) and false positive cases (Fig. 6b). AG-1 surveys the nodule and the microenvironment, AG-2 removes the attention on the nodule and surveys the surrounding microenvironment, and CAM at the last layer shifts the attention back to the nodule parenchyma. For true negative cases (Fig. 6c) and false negative cases (Fig. 6d), another attention pattern is observed. The network attention starts with AG-1 the nodule and the microenvironment. Sequentially at the AG-2 layer, the network removes the attention from the majority of the nodule parenchyma with a focus on the microenvironment. At the final stage, the network completely removes the attention from the nodule.

In summary, the network conducts similar search patterns at AG-1 and AG-2 stages for all the cases, then if the network attention shifts back to the nodule at the final stage, the classification will be malignant, while the attention remains at the microenvironment, the classification will be benign. In Fig. 6**.**, We demonstrate cases of four possibilities including true positive (a), false positive (b), true negative (c), and false negative (d). It also presents the attention-gated maps and CAM of each case from left to right, respectively. We found that false positive cases usually occurred with larger nodule sizes, especially when the nodule size exceeds our per-defined 64 × 64 × 64 ROI, therefore, there is limited nodule shape, boundary, and microenvironment information presented to the network. For false negative cases, nodules with less diffuse characteristics and more well-defined boundaries are more likely to be classified as benign by the network.

As for fibrosis classification, the network initially searches the surrounding soft tissue area close to the nodule and gradually enlarges the survey area in lung parenchyma to detect fibrotic tissues.

## Discussion

To evaluate the role of the microenvironment in nodule malignancy classification, we compared the model trained with nodule and microenvironment against the model trained with nodule alone (microenvironment removed). The statistical analysis showed a statistical significance (CI: 95%) that the performance of the method using not only the background information but also the semantic metadata was superior in accuracy (p-value = 0.0011) and sensitivity (p-value = 0.0013). The Bonferroni adjusted alpha coefficient was 0.01667. In addition, we found that although sensitivity decreased vastly by 15.46% after removing the nodule’s microenvironment, the specificity slightly increased by 2.6%, indicating that the detection ability of malignant nodules decreased when the microenvironment was removed. While benign nodule detection remained unaffected. Thus, the factors that contribute to a nodule's malignancy may be dependent on the nodule and its microenvironment.

An intriguing observation pertains to comparing fibrosis incidence rates in malignant and benign nodules. Malignant nodules exhibit a fibrosis incidence rate of 65%, while benign nodules demonstrate a rate of 35%, representing a 30% higher fibrosis rate in the former. To examine the relationship between fibrosis and malignancy, we purposely added semantic fibrosis data into the classifier right before the final fully connected layer of the classification model. The classifier is, therefore, forced to consider the fibrosis information when making the final prediction. When comparing the model trained with and without the fibrosis metadata, we found that sensitivity increases by 9.21% when semantic fibrosis information is available, and specificity slightly decreases by 0.91%. In other words, the ability to detect malignant nodules was enhanced when fibrosis data was presented to the network during training.

In this study, we utilized two distinct approaches to assess nodule malignancy: VDT and deep learning models (3D AG-Net). VDT serves as a well-established method for evaluating nodule behavior predicated upon growth rate analysis. This approach offers valuable insights into the pace of nodule size alteration. However, the precision of VDT is susceptible to fluctuations due to factors such as measurement techniques, nodule characteristics, and the threshold values employed to classify nodules as either rapidly expanding or stable. VDT, rooted in quantitative measurements, exhibits relative independence from extensive datasets for training, yet its simplicity constrains its ability to encapsulate intricate nodule attributes.

Conversely, deep learning models have exhibited the capacity to uncover subtle features that may elude conventional methodologies. The accuracy of these models hinges on the quality and diversity of the training data, the architectural design of the model, and the criteria used for performance assessment. Consequently, deep learning models offer a more advanced and comprehensive evaluation of lung nodules.

In our experimental approach, the VDT method yielded an accuracy of 62.63%, with a sensitivity of 56.52% and specificity of 65.48%. Notably, 3D AG-Net trained on nodule-only images, devoid of microenvironment information, achieved an accuracy of 75.61%, sensitivity of 50.00%, and specificity of 88.46%. It is of significance that this CNN, trained exclusively with nodule images, outperformed the VDT method by 12.98% in terms of accuracy.

Note that there are some limitations in the study. First, we labeled all shrinking-size nodules as benign (negative VDT values based on the modified Schwartz formula). This assumption could lead to underestimating the VDT prediction accuracy and sensitivity because nodules with a decreased size could still be cancer. Second, we used a segmentation model to segment lung nodules and then remove the background. Errors such as cases with partial surrounding tissue leaking into the dataset, or nodules partially removed by the segmentation algorithm, could propagate from the segmentation stage and affect the classification performance. Therefore, the performance without microenvironment could be over- or under-estimated due to the limitation of the segmentation model. It is worth considering the exploration of advanced segmentation models such as AMSUnet [26] and Dual-Branch-UNet [27] in future research endeavors.

## Conclusion

We have developed a DL-based pipeline containing an auto-segmentation model (3D U-net) and an attention-gated classification model (3D AG-Net) to classify lung nodule malignancy and pulmonary fibrosis. The model’s attention can be visualized at different depths thus making the model behavior interpretable. We have successfully demonstrated that the nodule microenvironment, especially fibrosis, contributes to the performance of the nodule malignancy classification model. Microenvironment data increases nodule malignancy classification accuracy, sensitivity, and AUC. Model Performance is further increased when semantic lung fibrosis information becomes accessible.

### Supplementary Information

**Additional file 1**: **Appendix SA.** How the nodule segmentation model is implemented on LIDC-IRDI and in-house dataset. **Appendix SB.** How Class Activation Maps (CAM) assist the prediction of our model. **Appendix SC.** Performance comparison with state-of-the-art methods with LIDC dataset.

## Data Availability

The data that support the findings of this study are available from the corresponding author upon reasonable request.

## Abbreviations

3D AG-Net: 3D Attention-gated Network
AUC: Area-under-curve
CAM: Class activation map
CNNs: Convolutional neural networks
CT: Computed tomography
DL: Deep learning
ROC: Receiver operating characteristic
VDT: Volume doubling time
VIA: VGG image annotator
