# Supplementary material for: Lung nodule malignancy classification with associated pulmonary fibrosis using 3D attention-gated convolutional network with CT scans
Source: J Transl Med. 2024 Jan 13;22:51. doi: 10.1186/s12967-023-04798-w (PMC10787502; doi:10.1186/s12967-023-04798-w)
Supplement: Supplementary file 1 — Additional file 1: Appendix SA. How the nodule segmentation model is implemented on LIDC-IRDI and in-house dataset. Appendix SB. How Class Activation Maps (CAM) assist the prediction of our model. Appendix SC. Performance comparison with state-of-the-art methods with LIDC dataset. [file 12967_2023_4798_MOESM1_ESM.docx]

**Appendix A: How the nodule segmentation model is implemented on LIDC-IRDI and in-house dataset.**

**Segmentation Model:**

Our 3D segmentation model (3D UNet, Monai (21)) based on U-Net architecture which comprises an analysis path, encoder, and a synthesis path, decoder processed 1088 cases to generate masks for nodules.Our model contains 3D convolution layers and 3D pooling layers to extract the input volume's expressive features. In the decoder part, 3D deconvolution is used to restore the feature map size. We implanted the enhanced version of UNet, which has residual units. These residual units allow the model to capture rich semantic information around the lung nodules. They ensure the entire network can be more accurate to improve image segmentation of lung nodules. Our model was trained using the Pytorch deep learning framework. The learning rate and dropout rate were adopted as 0.0001 and 0.2, respectively. We used the ADAM optimizer to train. Data augmentation, including random flips, intensity scaling, and intensity shifting, were used to improve model performance. Dice loss function was used as a metric to access the model performance and evaluate the testing dataset. The sliding window inference method which uses a rectangular or cube region of fixed dimension that "slides" across an image was applied to create binary classifications on whether or not each voxel belongs to a nodule. Although computationally expensive, this method determines if the window has an object that interests us. Accelerated methods such as cache IO and transform function featured by MONAI were also used to expedite the training process.

All experiments including preprocessing, developing, and evaluation of the model, were performed using Python version 3.6 and PyTorch 1.5 on NVIDIA GP102, GTX 1080 Ti.

**Nodule segmentation:**

We observed a smooth decrease in the training loss with a fluctuated mean dice score in the validation loss due to the overfitting of the model. Therefore, we include a dropout rate of 20% during each epoch. Example cases of the segmentation results are shown in Figure 3 for the LIDC dataset and the in-house dataset. We were able to see the similarity between the ground truth and the generated mask. We achieved an average dice score of 0.761 on the validation dataset after 50 epochs of training. The segmentation model is a stage of our pipeline that prepares inputs for nodule malignancy prediction. The model generates a dataset excluding the background information and a dataset including only the background information for prediction models. **Figure 3** (Columbia dataset) indicates a comparison of the original CT volume, the nodule cropped volume, and the background removed volume. Both nodules cropped volume and background removed volume were fed as training data to next step classification models.

**Nodule volume doubling time estimation:**

The nodule doubling time can be estimated through the trained segmentation model. Within all the dataset, we collected 288 study cases which have multiple scans on two timestamps. Unlike the clinical estimation, which uses a maximum diameter to gauge a 3D volume, we acquired precise volume estimation from the segmentation model. Furthermore, we estimate the VDT using the modified Schwartz formula. The VDT method achieved 62.6%, 43.3%, 56.5%, and 65.48% for accuracy, precision, sensitivity, and specificity, respectively, predicting nodule malignancy (**Table 2**). Note that the ROC curve is plotted by choosing a series of discrimination thresholds. Given a single discrimination threshold (VDT=500 days), the ROC curve is not achievable in our case.

**Appendix B: How Class Activation Maps (CAM) assist the prediction of our model.**

The attention map is a color-coded map of attention coefficients (Eq. 5), which highlights the task-relevant features on the images [[25]](https://paperpile.com/c/lp8XF0/LvxcG). Attention coefficients ($\alpha$) can be computed as follow: we denote a set of vectors of features (f) and dimensions (d) as $V\in R^{f \times d}$

$\alpha^{l}=Sigmoid\left[ V^{T}\left( ReLU\left( V_{\left( x \right)}^{T}x^{l}+V_{\left( g \right)}^{T}g^{\left( l+1 \right)} \right) \right) \right]$(5)

Where $x^{l}$ is the feature vector of length $f_{x}$In the activation map of a specific layer $l$; $g$is the global feature vector taken from $l+1$layer (one layer deeper) next to $x$, it has smaller dimensions and better feature representation. The linear transformations are defined as $V_{x}^{T}\in R^{f_{x}\times d_{x}/2},$ which is a strided convolution reduce $x$ dimensions to half; $V_{g}^{T}{\in R}^{{2f}_{g}\times d_{g}}$is a 1 x 1 x 1 convolution such that $f_{g}$doubled. After linear transformations, vectors $g$ and $x$ are aligned ($f_{x}= 2f_{g}; d_{x}/2 = d_{g}$) and summed element-wise. The additive process results in emphasizing aligned weights while diminishing unaligned weights. The summed vectors then pass through ReLU activation layer and $V^{T}{\in R}^{1 \times d_{g}}$ that collapses the features into 1. The values are further restricted to [0, 1] by the Sigmoid function. Eventually, attention coefficients at layer, $\alpha^{l},$ can be computed by trilinear interpolation upsampling to match $d_{x}$. Along with attention maps, CAMs generated by global average pooling at the final layer of the network are also used to visualize the network behavior [[28]](https://paperpile.com/c/lp8XF0/U34vm). CAMs highlight the discriminative images regions used by the whole network, therefore, help us to understand the prediction made by the network.

**Appendix C: Performance comparison with state-of-the-art methods with LIDC dataset.**

Each nodule was rated from 1 to 5 where a score of 1 indicates "Highly Unlikely to be Malignant," S2 "Moderately Unlikely to be Malignant," S3 "Indeterminate Likelihood," S4 "Moderately Suspicious for Malignant," and S5 "Highly Suspicious to be Malignant." As proposed by [[29]](https://paperpile.com/c/lp8XF0/HEqwH), scores of 1, 2, 3 were considered benign, and 4, 5 were considered malignant. Most recent studies excluded S3 nodules because S3 nodules are of ambiguous malignancy [[11]](https://paperpile.com/c/lp8XF0/iMTY5), [[12,30]](https://paperpile.com/c/lp8XF0/mNQZ2+Nd1AN), [[15]](https://paperpile.com/c/lp8XF0/2F41X). However, removing S3 nodules could result in biased models with artificially boosted performance. Therefore, this study used the complete LIDC dataset to produce a more generalized prediction model to better transfer pre-trained weights onto the in-house dataset. As for the model performance comparison with the state-of-the-art methods, we excluded the S3 nodules, using S1, S2, S4, and S5 to coincide with the methodology.

To validate the effectiveness of our 3D AG-Net, we observed the model performance using the LIDC dataset against other methods in the literature. We followed previously described methodologies to remove nodules with average scores of 3 and regard nodules with average score <3 and >3 as benign and malignant ones[[29]](https://paperpile.com/c/lp8XF0/HEqwH)[[31]](https://paperpile.com/c/lp8XF0/jYJkm). Shen et al. [[13]](https://paperpile.com/c/lp8XF0/9xfOb) proposed a 3D deep hierarchical semantic convolutional neural network (HSCNN) with 4-fold cross-validation to predict semantic features such as calcification, margin, texture, sphericity, subtlety along with nodule malignancy to explain how the model interprets images. Zhu et al. [[11]](https://paperpile.com/c/lp8XF0/iMTY5) used a 3D deep dual-path network (DPN), and Ziao et al. [[12]](https://paperpile.com/c/lp8XF0/mNQZ2) conducted an ensemble learning to combine denoising autoencoder, ResNet-18, texture, and shape features. Al-Shabi et al. [[14]](https://paperpile.com/c/lp8XF0/iTmdD) proposed Local-Global networks to extract local features and global features for nodule classification with 10-fold cross-validation. Hu et al. used an attention-guided differentiable architecture search technique to refine the classification performance [[15]](https://paperpile.com/c/lp8XF0/2F41X). The 3D AG-Net used in this study achieved a consistent performance with the above-discussed strategies for nodule classification on the LIDC dataset.

| **Benign/Malignant** | **Method** | **2D/3D** | **Accuracy** | **sensitivity** | **Precision** | **AUC** |
| --- | --- | --- | --- | --- | --- | --- |
| S123/S45 | HSCNN  Shen et al. [[13]](https://paperpile.com/c/lp8XF0/9xfOb) | 3D | 84% | 71% | - | 87% |
| S12/S45 | DeepLung  Zhu et al. [[11]](https://paperpile.com/c/lp8XF0/iMTY5) | 3D | 88.74% | - | - | - |
| S12/S45 | Ensemble  Xiao et al. [[12]](https://paperpile.com/c/lp8XF0/mNQZ2) | 2D | 93.1% | 81.75% | 83.85% | 82% |
| S12/S45 | Local-Global  Al-Shabi et al.[[14]](https://paperpile.com/c/lp8XF0/iTmdD) | 2D | 88.46% | 88.66% | 87.38% | 96% |
| S12/S45 | A-DARTS  Hu et al. [[15]](https://paperpile.com/c/lp8XF0/2F41X) | 2D | 92.93% | 84.38% | - | 96% |
| S12/S45 | Ours | 3D | 91.57% | 83.34% | 90.46% | 95% |
